# Importance of confirmatory test characteristics in optimizing community-based screening for tuberculosis: an epidemiological modeling analysis

**DOI:** 10.1186/s12879-025-11905-3

**Published:** 2025-10-27

**Authors:** Lukas E. Brümmer, Theresa S. Ryckman, Sourya Shrestha, Florian M. Marx, William Worodria, Devasahayam J. Christopher, Grant Theron, Adithya Cattamanchi, Claudia M. Denkinger, David W. Dowdy, Emily A. Kendall

**Affiliations:** 1 Department of Infectious Disease and Tropical Medicine, Heidelberg University Hospital, Heidelberg, Germany; 2 German Center for Infection Research (DZIF), Partner site Heidelberg, Heidelberg, Germany; 3 Department of Epidemiology, Johns Hopkins Bloomberg School of Public Health, Baltimore, MD USA; 4 Center for Tuberculosis Research, Division of Infectious Diseases, Johns Hopkins University School of Medicine, Baltimore, MD USA; 5 Department of Medicine, Mulago National Referral Hospital, Kampala, Uganda; 6 Department of Pulmonary Medicine, Christian Medical College, Vellore, India; 7 DSI-NRF Centre of Excellence for Biomedical Tuberculosis Research, Division of Molecular Biology and Human Genetics, Faculty of Medicine and Health Sciences, Stellenbosch University, Cape Town, South Africa; 8 South African Medical Research Council Centre for Tuberculosis Research, Cape Town, South Africa; 9 Division of Pulmonary Diseases and Critical Care Medicine, University of California Irvine, Irvine, CA USA; 10 Center for Tuberculosis, Division of Pulmonary and Critical Care Medicine, University of California San Francisco, San Francisco, CA USA; 11 Im Neuenheimer Feld 324, 69120 Heidelberg, Germany

**Keywords:** Tuberculosis, Active case-finding, Confirmatory testing, Epidemiological modeling

## Abstract

**Background:**

The costs, operational barriers, and sensitivity of available tools to confirm a TB diagnosis limit current active case-finding (ACF) efforts for tuberculosis (TB). However, it is not well understood which of these limitations have the greatest epidemiological relevance and might therefore warrant prioritization in confirmatory test development.

**Methods:**

To explore which features of the confirmatory testing step most influence the impact of ACF, we developed a state-transition model of a one-time, community-based ACF campaign, with a fixed budget of one million United States dollars for screening and confirmatory testing, assuming an adult target population with four times the national prevalence of Uganda. We compared TB diagnoses, mortality, and transmission when conducting ACF with a currently available confirmatory test (mirroring sputum-based Xpert Ultra) versus ACF with an improved confirmatory test, i.e., (1) increased sensitivity (from 69% to 80%), (2) non-sputum specimen type (increasing specimen availability from 93% to 100%), (3) immediate turn-around of test results at the point-of-care (increasing delivery of positive results from 91% to 100%), or (4) reduced costs (from $20 to $10 per confirmatory test).

**Results:**

In a simulated target population of 500,000 adults, 8,029 (1.6%; 95% uncertainty range [UR] 6,634-9,380) had TB disease, and 1,136 (789-1,586) were projected to die of TB in the absence of ACF. Assuming current tests, ACF could reach 149,811 (90,834 − 217,928; 30% of the target population) people under the allotted budget, connecting 1,151 (676-1,813) individuals with TB to treatment and averting 135 (64–249) deaths. Higher diagnostic sensitivity most increased the number of people with TB who received treatment as a result of ACF (by 15% [5–27%]). However, improvements that could benefit individuals regardless of their sputum bacillary load, such as reduced test costs, achieved larger reductions in mortality (11% [4–36%]).

**Conclusion:**

Due to greater detection of individuals with high bacillary load, making confirmatory tests for community-based TB screening less expensive and more accessible may lead to greater population health benefits than further increasing test sensitivity. Nonetheless, achieving large (> 20%) increases in the health impact of ACF will require improvements to components of ACF other than the confirmatory diagnostic test.

**Supplementary Information:**

The online version contains supplementary material available at 10.1186/s12879-025-11905-3.

## Introduction

Every year, an estimated four million people with new tuberculosis (TB) are not notified to public health authorities and many of these people are never diagnosed or treated [1]. Community-based screening or active case-finding (ACF) can potentially reduce TB mortality and transmission potential, by detecting and linking people with TB to treatment that would have otherwise been missed or only diagnosed after they have transmitted TB to others [2]. The uptake and epidemiological impact of ACF, however, is limited by deficiencies of currently available diagnostic tests [3].

Most ACF algorithms begin with a low-cost screening test or symptom survey, and thus require a second, more specific test to confirm positive screening results [4]. Currently, advanced tools for confirmatory testing include molecular sputum tests such as Xpert Ultra MTB/RIF (Cepheid, Sunnyvale, CA, United States; “Xpert Ultra”) [5–7], but even the best tools available are subject to several shortcomings. First, high testing costs [8] may limit the number of people who can be tested under a given budget. In addition, the need for a sputum specimen hampers testing reach. Furthermore, the long turn-around times associated with off-site and high-volume testing can inhibit people from receiving their test results [6]. Moreover, even molecular tests designed for high sensitivity leave some individuals with low bacillary load undetected [9].

The most recent target product profile published by the World Health Organization (WHO) for TB diagnostics highlights the critical need to improve diagnostic tools for TB, particularly through the development and use of non-sputum specimens and enabling point-of-care testing [10]. Many of the TB diagnostics development efforts that are underway could lead to tools that would overcome these shortcomings. However, it is not clear which test characteristics could provide the largest benefit when used for the confirmatory step in ACF algorithms. For example, particularly for the objective of reducing transmission, it might be more impactful to improve detection of individuals who have high respiratory bacterial TB burden but who are missed due to other barriers than to detect TB with bacterial burdens below the limit of detection of current diagnostics. If so, it could be more critical for test development to reduce operational barriers or test costs during test development than further increasing test sensitivity.

To guide researchers and public health decision makers in developing valuable tools for confirming a TB diagnosis within an ACF campaign, we developed a model to evaluate the impact of advancing key characteristics of confirmatory testing within the context of community-based screening for TB. We consider an illustrative community-based ACF campaign that begins with chest X-ray screening in a high-TB-risk population in a setting similar to Uganda. We then estimate the impact of ACF on TB mortality, transmission potential and treatment initiation, when using Xpert Ultra versus various enhanced, hypothetical assays as the confirmatory test.

## Methods

### Model design and TB disease course

**Fig. 1:**
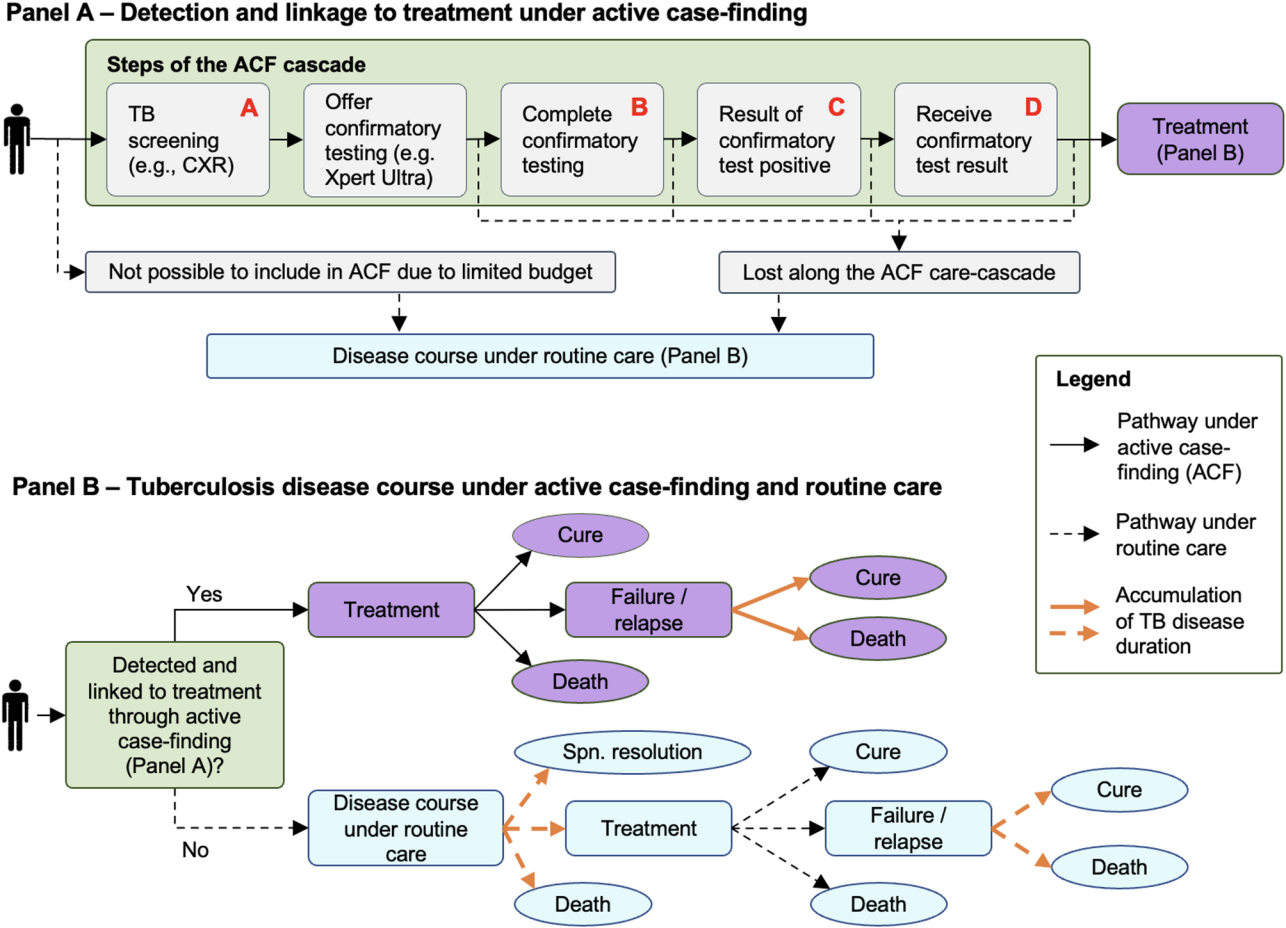
Model structure. We model a one-time active case finding (ACF) campaign, consisting of one-time screening (modeled as chest X-ray) plus confirmatory testing for those who screen positive (Panel A). We consider four different improvements to the confirmatory test that could increase the number of people successfully diagnosed and treated under ACF efforts: (A) reduced testing costs from $20 to $10 per test; (B) a non-sputum specimen type, increasing confirmatory specimen availability from 93% to 100%; (C) a point-of-care format, increasing successful delivery of confirmatory test results from 91% to 100%; (D) increased test sensitivity from 69% to 80%. Improvement A increases the number of people that can be included in the ACF efforts under a budget constraint, while improvements B, C, and D directly enhance the confirmatory testing process (Table 1). We estimate TB disease outcomes under ACF or – for people with TB who are not screened or not detected through ACF – under routine care using a decision-tree model, projecting TB to eventually end in cure, spontaneous resolution, or death (Panel B). The modeled transition probabilities within both the ACF care cascade (Panel A) and the future TB disease course (Panel B) depend on an individual’s symptom, smear, and HIV status at the time of the intervention. In addition, we project smear-negative and smear-positive disease duration until any of these endpoints are reached (depicted through the bold, orange arrows in Panel B)

To estimate the epidemiological impact of improving different characteristics of the TB confirmatory test used in ACF, we construct a state-transition model of a one-time ACF campaign for adults, conducted on a background of routine TB care (Table 1). The results of ACF (detected and linked to treatment, or not) are estimated by applying probabilities that individuals receive the screening test, are offered and complete confirmatory testing, test positive, receive their result, and link to treatment (Fig. 1A). We next place these steps within a larger decision tree to project individual-level clinical outcomes as well as the cumulative duration of TB disease under routine care and with the addition of ACF (Fig. 1B). We differentiate prevalent TB, and associated state-transition probabilities, by three characteristics: (i) high- or low-bacillary load (corresponding to positive or negative smear-microscopy status), (ii) presence of chronic cough (“cough positive” if a person with TB would report cough for more than two weeks, or “cough negative” otherwise), and (iii) human immunodeficiency virus (HIV) infection status (“HIV positive” or “HIV negative”). We assume that people with high bacillary loads are four-times more infectious (based on molecular epidemiology studies [11] and also supported by contact infection studies [12, 13]) and that they are more likely to test positive with any given confirmatory test (estimated for Xpert Ultra from diagnostic accuracy studies [4, 9]). As an illustrative setting, we consider a hypothetical population of 500,000 adults in Uganda (“target population”) living in a geographic area that is targeted for screening based on estimated TB risk. Based on geographical variability in TB burden estimates [14], we model the target population as having a TB prevalence of four times the national average (1.6%) [15] with a joint distribution of TB bacillary load, HIV status, and chronic cough reflecting nationwide patterns [15–18] (Table 2).

**Table 1:** Hypothetical improvements of a test to confirm TB during active case-finding efforts

		Test characteristic				Mechanism of benefit
		Test costs in 2025 USD (95% uncertainty range)	Specimen type	Delivery of test result	Test sensitivity (95% uncertainty range)	
Test improvement modeled	Baseline test	20 (16 to 26) [8]	Sputum	After 2–3 days	69% (48 to 86%) [4, 19, 45]	--
	A) Test with reduced test costs	50% of the baseline value [35]^a^	Sputum	After 2–3 days	69% (48 to 86%) [4, 19, 45]	Reduced test costs (including cartridge-costs, labor, equipment, consumables) allow more people to be screened under the same budget.
	B) Test with a non-sputum specimen	20 (16 to 26) [8]	Non-sputum ^b^	After 2–3 days	69% (48 to 86%) [4, 19, 45]	Using a universally available non-sputum specimen increases completion of testing by including those who cannot produce sputum.
	C) Test with immediate turn-around of test result	20 (16 to 26) [8]	Sputum	Immediately at the point-of-care ^c^	69% (48 to 86%) [4, 19, 45]	Reducing the time to result, as with a point-of-care test completed in 15 min or less, reduces loss due to unsuccessful delivery of confirmatory test results.
	D) Test with increased sensitivity	20 (16 to 26) [8]	Sputum	After 2–3 days	80% (80 to 80%) [35]^d^	Fewer false-negative test results means that more people with TB receive a positive confirmatory test result.

*TB* tuberculosis, *USD* United States dollar

^a^ Assumes consumables costs to be reduced from $8 to $4 (similar to the optimal target for a rapid, sputum-based test in the 2014 World Health Organization Target Product Profile [35]), with proportional reduction in labor/equipment costs

^b^ Modeled as increasing the proportion of prevalent TB with abnormal CXR completing confirmatory testing from 93% (95% uncertainty range [UR]: 89 to 96%; see Table 2) to 100% (100 to 100%)

^c^ Modeled as increasing the proportion of prevalent TB that has completed confirmatory testing successfully receiving the confirmatory test result from 91% (84 to 96%) [6] to 100% (100 to 100%)

^d^ Corresponds to the minimal sensitivity requirement for a rapid, non-sputum-based test to diagnose TB at the microscopy center level as per the 2014 World Health Organization Target Product Profile [35]

**Table 2:** Key model parameters

Parameter	Estimate (95% uncertainty range)	Distribution	Source
Baseline population estimates, assuming a population of 500,000 individuals ^a^			
Number of individuals with bacillary load (↑) TB, HIV (+), chronic cough (+)	407 (211 to 696)	See Appendix, Table S1, for underlying assumptions	
Number of individuals with bacillary load (↑) TB, HIV (+), chronic cough (-)	508 (238 to 917)		
Number of individuals with bacillary load (↑) TB, HIV (-), chronic cough (+)	1,374 (955 to 1,880)		
Number of individuals with bacillary load (↑) TB, HIV (-), chronic cough (-)	976 (361 to 1,627)		
Number of individuals with bacillary load (↓) TB, HIV (+), chronic cough (+)	897 (580 to 1,313)		
Number of individuals with bacillary load (↓) TB, HIV (+), chronic cough (-)	1,127 (628 to 1,746)		
Number of individuals with bacillary load (↓) TB, HIV (-), chronic cough (+)	1,239 (771 to 1,770)		
Number of individuals with bacillary load (↓) TB, HIV (-), chronic cough (-)	1,402 (670 to 2,201)		
Number of individuals without TB, HIV (+), chronic cough (+)	8,063 (6,692 to 9,462)		
Number of individuals without TB, HIV (+), chronic cough (-)	17,957 (15,743 to 20,135)		
Number of individuals without TB, HIV (-), chronic cough (+)	20,692 (19,154 to 22,255)		
Number of individuals without TB, HIV (-), chronic cough (-)	445,266 (442,959 to 447,545)		
TB disease outcomes under routine care (average across all prevalent TB) ^b^			
Proportion of prevalent TB ending in death	14% (10 to 19%)	See Appendix, Table S10, for further details, including differences in outcomes by individual characteristics, and Appendix, Text S3 for underlying assumptions	
Proportion of prevalent TB ending in cure following treatment	38% (31 to 46%)		
Proportion of prevalent TB resolving spontaneously	47% (40 to 55%)		
Accuracy of chest X-ray and sputum-based Xpert Ultra (average across all prevalent TB) ^c^			
Chest X-ray – sensitivity	90.0% (84.9%* to 94.1%*)	Beta	[15]
Chest X-ray – specificity	96.0% (93.0% to 97.0%)	Beta	[4]
Sputum-based Xpert Ultra – sensitivity	69.0% (48.0% to 86.0%)	Beta	[4, 19, 45]
Sputum-based Xpert Ultra – specificity	98.8% (97.2% to 99.5%)	Beta	[4]
Gaps in the TB care-cascade			
Proportion of individuals with chronic cough unable to produce sputum	4.8%^d^ (1.5%^d^ to 14.2%^d^)	Beta	[20]^d^
Proportion of individuals without chronic cough unable to produce sputum	8.0%^d^ (3.7%^d^ to 16.6%^d^)	Beta	[20]^d^
Proportion of ACF target population not receiving confirmatory test result	9.4% (4.2% to 15.8%)	Beta	[6]
Proportion not offered treatment despite having received a positive confirmatory test result	9.3% (9.2% to 9.4%)	Beta	[22]
Costs estimates (2025 USD)			
Total costs to screen one person (using chest X-ray)	5 (4 to 7)	See Appendix, Text S2 and Table S3 for underlying assumptions	
Total costs to perform confirmatory testing on one person (using sputum-based Xpert Ultra)	20 (16 to 26)		
Total costs to treat one person for TB ^e^	169 (80 to 258)	Gamma	[52]

* Lower and upper bounds were estimated from the published point estimate and sample size, using the qbeta function from the R base packages

^a^ The sum of all people with smear positive TB, all people with smear negative TB, and all people without TB does not equal the size of the total population, due to uncertainties in values introduced through the Monte Carlo simulation

^b^ Values stratified by individual characteristics are provided in appendix, Table S10

^c^ Values stratified by individual characteristics are provided in appendix, Table S2

^d^ These proportions are based on data from Klinkenberg et al. [20] and an ongoing clinical trial (Clinic-based Versus Hotspot-focused Active TB Case Finding [CHASE-TB], ClinicalTrail.gov ID: NCT05285202) [21]. Results from both studies were pooled using the *metaprop* function (package “meta”) in R version 4.0.2

^e^ Only relevant in the scenario analysis including TB treatment costs as part of the active case-finding budget

### Active case-finding

We model an ACF campaign consisting of screening for TB in high-risk communities, followed by confirmatory testing for those who screen positive. To reflect the current best available tools, we assume that the ACF campaign uses mobile chest X-ray to screen adults for TB, with immediate artificial intelligence-based reporting of results [4, 15, 19]. For people screening positive, we assume confirmatory testing to be immediately attempted, using sputum-based Xpert Ultra as the diagnostic test in the base case [5–7], or using a variety of hypothetical confirmatory tests in comparative analyses of the impact of confirmatory-test improvements (see section ‘Confirmatory test improvements’ below). When using Xpert Ultra, individuals unable to produce sputum are excluded from confirmatory testing; sputum production is estimated based on systematic TB screening efforts modeling people with chronic cough as more likely to produce sputum than those without chronic cough [20, 21]. We assume that the time taken to complete confirmatory testing results in some individuals being lost from the screening cascade before delivery of confirmatory test results (e.g., because samples are lost in transit or because health workers are unable to contact them to inform them of positive results). Those who receive and are informed of positive results are referred to treatment services, although, based on other modeling estimates [22], some additional losses during linkage to treatment are accounted for regardless of the confirmatory test. For participants who initiate treatment as a result of ACF, outcomes following treatment are allocated such that they are not worse (i.e., increased TB mortality or longer disease duration) than under routine care (Tables 2 and Appendix, Text S1) [23–26].

### Costs of active case-finding and budget constraint

In order to compare various confirmatory test improvements, we consider an arbitrary limited budget of one million United States Dollar (USD) for the ACF effort, assuming that some of the target population will not be screened due to budget constraints. This budget includes screening and confirmatory testing costs, but we assume treatment costs for people diagnosed with TB to be budgeted separately; the latter assumption is modified in sensitivity analysis. For each modeled confirmatory test, we estimate the number of people who could be screened under the available screening and testing budget, based on the per-participant cost of the screening step (including staff, transport, and screening test costs), the proportion of participants who require confirmatory testing, and the per-person cost of confirmatory testing. Costs are derived from local procurement costs and implementation studies (Table 2 and Appendix, Text S2 and Table S3) [8, 27–33]. All costs are presented in 2025 USD (inflated using World Bank consumer price indices where applicable [34]) and from the healthcare system perspective.

### Confirmatory test improvements

Our primary comparison is between ACF utilizing Xpert Ultra on an expectorated sputum specimen (“baseline confirmatory testing”) [4, 9] and a series of ACF campaigns with hypothetical improved confirmatory tests, each of which improves on the background of Xpert Ultra in one aspect (further details in Fig. 1; Table 1): (A) reduced test cost, (B) non-sputum specimen, (C) immediate turnaround, and (D) increased test sensitivity. The magnitudes of improvements to cost and sensitivity were based on optimal target product profiles [35], and improvements to the specimen type and the turn-around time were assumed to eliminate losses associated with inability to produce sputum and with the time required to complete confirmatory testing, respectively, in the current care cascade. While some of the confirmatory test improvements modeled reflect current advances in test development, e.g., replacing sputum-specimens through oral-swab based testing (below), most represent aspirational benchmarks that might be achieved in the future.

### Oral-swab based testing

One diagnostic testing strategy currently in development is oral-swab based testing. Oral swabs might be a more readily available specimen but result in reduced sensitivity (existing prototypes have been estimated to have sensitivities between 52% and 97% relative to sputum molecular testing [36–38]). To reflect the potential impact of this testing method, we also project the minimal sensitivity required for oral-swab-based confirmatory testing to achieve at least the same epidemiological impact as the baseline confirmatory test. We assume oral-swab based testing to be available at 50% of the costs of sputum-based Xpert Ultra and to allow for near point-of-care testing, increasing the proportion of individuals receiving their test result to 100% [38]. We assume that incremental false negatives occur in individuals with low bacillary load first [37] and that oral-swab sensitivity is independent of ability to expectorate except as mediated by the presence of chronic cough. To account for uncertainty in the proportion of people able to expectorate sputum for confirmatory testing, we perform these analyses at values of 93% (as in the primary analysis), 83%, and 73% for the proportion of people able to submit sputum (Appendix, Text S5).

### TB diagnosis and treatment under background routine care

For people with TB not included in ACF efforts – due to either budget constraints or losses along the ACF cascade – outcomes of routine care are determined by our model. Under routine care, an episode of TB can end before any treatment, either through death or through spontaneous resolution. As a second option, treatment can be initiated, in which case the disease course can end in either eventual cure or, for some whose treatment is unsuccessful, TB death after treatment (Fig. 1).

We estimate the competing probabilities of dying or experiencing spontaneous TB resolution before any treatment under routine care versus receiving treatment under routine care based on results from a published smear- and symptom-stratified model of TB natural history [12]. This model – which accounts for transitions of smear and symptom status over time and aligns survival outcomes of untreated TB with empirical data from the pre-antibiotic era and also simulates outcomes in the context of present-day clinical services – estimates that a high proportion of people with smear-negative TB will experience spontaneous TB resolution. Given that approximately 60% of people with prevalent TB in our modeled setting have a low bacillary load at any given time, our model similarly projects that a high proportion of prevalent TB (47% [95% uncertainty range (UR): 40–55%]) will spontaneously resolve. We also conduct a sensitivity analysis assuming no spontaneous resolution of TB prior to receiving treatment (described in detail in a later section). We use the same model’s estimates of disease duration to estimate the time that each person spends with untreated TB, separately estimating the cumulative amounts of time with high and with low bacillary loads (Appendix, Text S3 and Table S5). With the model providing estimates only for HIV-negative individuals with TB, for HIV-positive individuals, we adjust these estimates using results from another model of TB disease courses that incorporated HIV-stratified estimates of TB notification and mortality; in the absence of data for Uganda, we apply estimates for Kenya [39–41] (Appendix, Text S3 and Table S5).

For people with TB who start treatment through routine care, the probability of an eventual outcome of cure versus TB death is projected based on a combination of treatment outcomes as reported to the WHO plus clinical trial results to estimate relapse risk (Appendix, Text S3, Table S4, and Table S6). Estimates are of the eventual outcome of a TB episode, after any retreatments that may be required due to failure or relapse [23–26, 42, 43]. In estimating how relapse and failure contribute to the cumulative duration of TB, we assume that the average time spent with TB after an initial recurrence is equal to the average duration of TB prior to any treatment, after stratification by HIV status.

### Outcome measures and reporting

Our primary comparisons are of the health benefit of ACF, comparing ACF with the baseline confirmatory test to ACF that uses the improved confirmatory tests described above, when both are evaluated under the same constrained budget (Fig. 1). We estimate the health benefit of ACF using the following measures:

1. the total number of people linked to treatment through ACF,
2. the number of TB-related deaths averted through ACF, and.
3. the TB transmission potential averted through ACF (estimated as a difference in total high bacillary-load-equivalent person-months, adjusting for an estimated fourfold lower infectivity during time spent with low bacillary load [11]).

We then estimate the incremental change in each of these measures when the confirmatory test is improved, on absolute scales and relative to the impact achieved with the baseline test.

### Analysis and reporting

To capture uncertainty, we first simulate 10,000 iterations of the targeted community-based cohort of 500,000 adults and their disease courses under routine care; within each cohort, we then simulate all modeled diagnostic tests and perform pairwise comparisons. All parameters are independently sampled from beta (if bounded above by one) or gamma (if no upper bound) distributions reflecting the uncertainty in available primary data or published estimates (Table 2 and Appendix, Tables S1-6, with further details in Text S4). For each outcome, we report the median value across these simulated cohorts as the point estimate, with a 95% uncertainty range (UR) based on the 2.5th and 97.5th percentiles across all simulations. All analyses use R version 4.0.2 (R Foundation for Statistical Computing, Vienna, Austria). Ethical approval was not sought for this study as there was no human subject participation.

### Sensitivity and scenario analyses

We analyze one-way sensitivity of model results to setting-dependent variation in TB and HIV prevalence and diagnostic testing costs [19, 44–51] (Appendix Text S5 and Table S7). We also evaluate how results change in scenario analyses that (1) include treatment costs as part of the ACF budget (to assess the potential impact of improved confirmatory test specificity) [52], (2) assume that prevalent TB does not spontaneously resolve (while assuming that treatment initiations and deaths prior to treatment occur in the same ratio as in the base model), (3) model costs [53] and accuracy of screening by chronic cough rather than by chest X-ray, (4) assume that a smaller [54] proportion of individuals is able to expectorate sputum, (5) assume a smaller [54, 55] proportion of individuals is successfully linked to care, and (6) model all confirmatory test improvements to be implemented simultaneously (Appendix Text S5 and Table S8).

## Results

### Population estimates

**Fig. 2:**
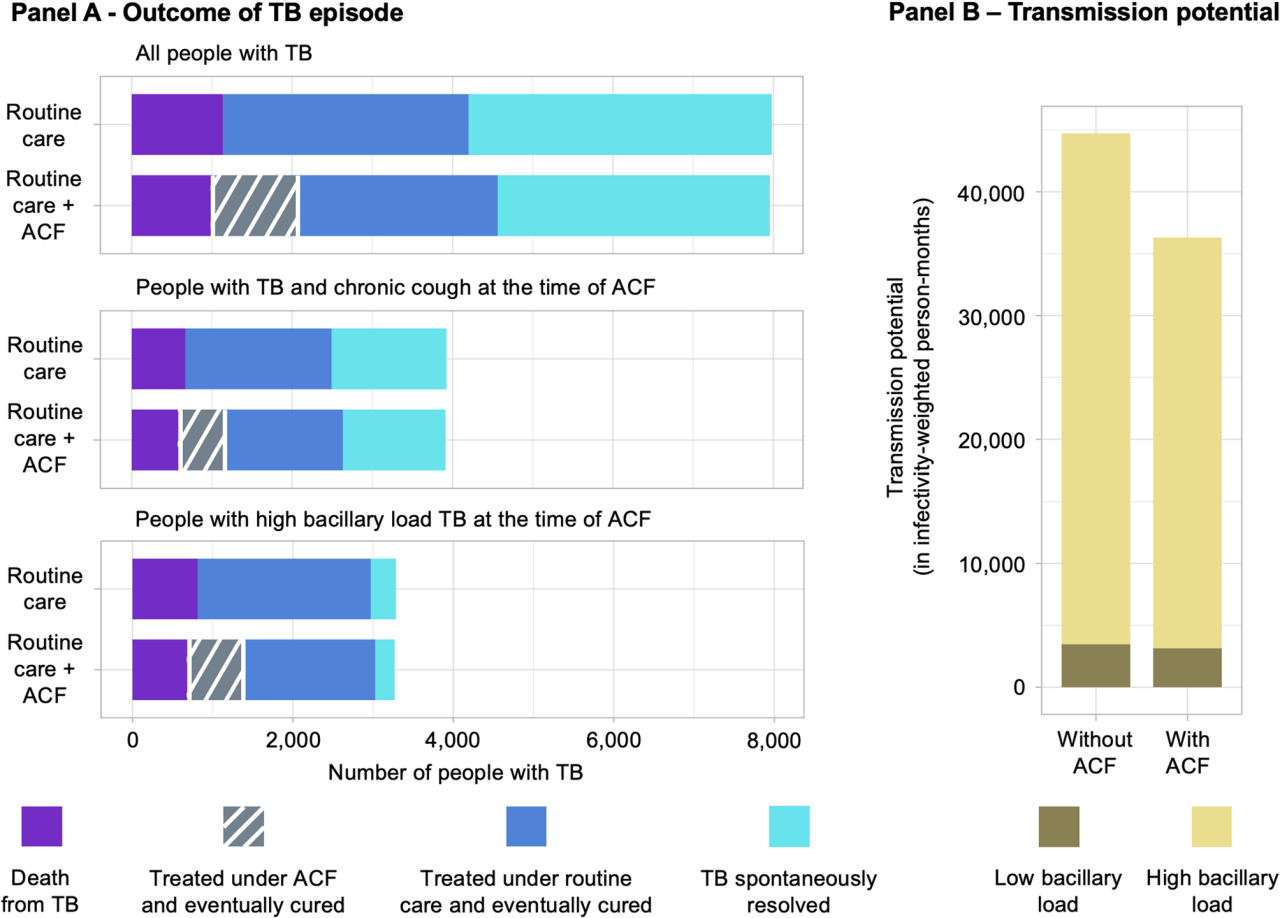
Projected epidemiological impact of a one-time active case-finding effort for tuberculosis in a high-burden population of 500,000 adults. Bars depict the estimated epidemiological impact of active case-finding (ACF) for tuberculosis (TB) among adults using Xpert Ultra as confirmatory test, compared to a situation where no community-based screening is in place (assuming a setting similar to Uganda). Panel **A** shows the number of adults with TB (top), with TB and chronic cough (middle), and with high TB bacillary load (bottom) that are projected to die from TB (purple), be treated and cured from TB (grey or dark blue) and in whom TB might spontaneously resolve or be cured in subsequent treatment attempts (light blue). The dark blue refers to the number of people that are linked to treatment through routine care efforts, i.e., symptom-based passive case-finding, and grey to those receiving treatment under ACF efforts. Panel **B** shows the future transmission potential (in infectivity-weighted person months) resulting from people with prevalent TB with high bacillary load (i.e., where smear microscopy would be positive; light yellow) and low bacillary load (i.e., where smear microscopy would be negative; dark yellow) TB, when ACF is not in place (left) versus when community-based ACF is conducted (right)

Our model projected 8,029 (95% uncertainty range: 6,634-9,380) people with TB in the target population of 500,000 adults, reflecting the assumed 1.6% (1.3–1.9%) prevalence of TB. Of the people with TB in the target population, 3,391 (2,588-4,330; 42% [35–50%]) people were projected to receive treatment through routine care in absence of any ACF. After estimating relapse risks and retreatment outcomes after treatment completion or loss to follow-up, 3,065 (2,339-3,914; 90% [89–92%]) of those who initiated treatment were projected to eventually be cured of TB. An estimated 1,136 (789-1,586; 14% [10–19%] of all people with TB) individuals were projected to die from TB in absence of ACF: 808 (484-1,241; 71% [58–81%]) before receiving treatment and 325 (237–435; 29% [19–42%]) afterwards. The cumulative duration of culture-positive and potentially infectious TB among this population in absence of ACF was 44,978 (29,013–66,784) infectivity adjusted person-months (Fig. 2).

### Community-based screening using Xpert ultra

Accounting for a constrained budget of $1 million, and estimating costs of $5 ($4–7) per person for chest X-ray screening and $20 ($16–25) for confirmatory testing (Table 2 and Appendix Text S2), we estimated that the ACF budget would allow 149,811 randomly selected individuals (90,834 − 217,928; 30% [18–44%] of the target population of 500,000 individuals) to undergo one-time TB screening. During this ACF campaign, TB would be detected in 1,270 (746-1,999) people, with 1,151 (676-1,813; 91% [91–91%]) of those started on treatment and 1,077 (633-1,702; 85% [84–86%]) eventually cured.

Of the people treated and cured as a result of ACF efforts with the baseline confirmatory test, 135 (64–249) were individuals who would have died had they not been detected through ACF. Thus, ACF reduced projected TB deaths in the target population to 996 (701-1,389), a 12% (7–19%) reduction compared to 1,136 (789-1,586) deaths without the ACF campaign. Furthermore, community-based ACF was projected to reduce future TB transmission potential to 36,496 (23,456 − 55,063) infectivity-adjusted person-months – a 18% (11–27%; 8,169 [4,182 − 15,020] infectivity-adjusted person-months) reduction of future TB transmission potential from the people with prevalent TB in the absence of ACF (Fig. 2).

### Impact of confirmatory test improvements

Of the test improvements modeled, increased sensitivity led to the largest incremental increase in TB treatment initiations (15% [5–27%] more people than through ACF with baseline confirmatory testing, 171 [57–349] additional people among the 149,811 individuals screened). This was followed by reduced test costs (11% [4–36%]; 135 [44–331]), immediate turn-around (11% [5–18%]; 121 [54–241]), and using a non-sputum specimen (8% [4–12%]; 87 [42–164]). By contrast, when only considering individuals with high bacillary load, the largest incremental increase in TB treatment initiations resulted from reduced test-costs (11% [4–36%] more people with high bacillary load than through ACF with baseline confirmatory testing; 86 [28–209] additional people), followed by immediate turn-around (11% [5–18%]; 77 [34–154]), using a non-sputum specimen (8% [4–12%]; 55 [26–104), and increased test sensitivity (1% [0–2%]; 7 [3–16]).

**Fig. 3:**
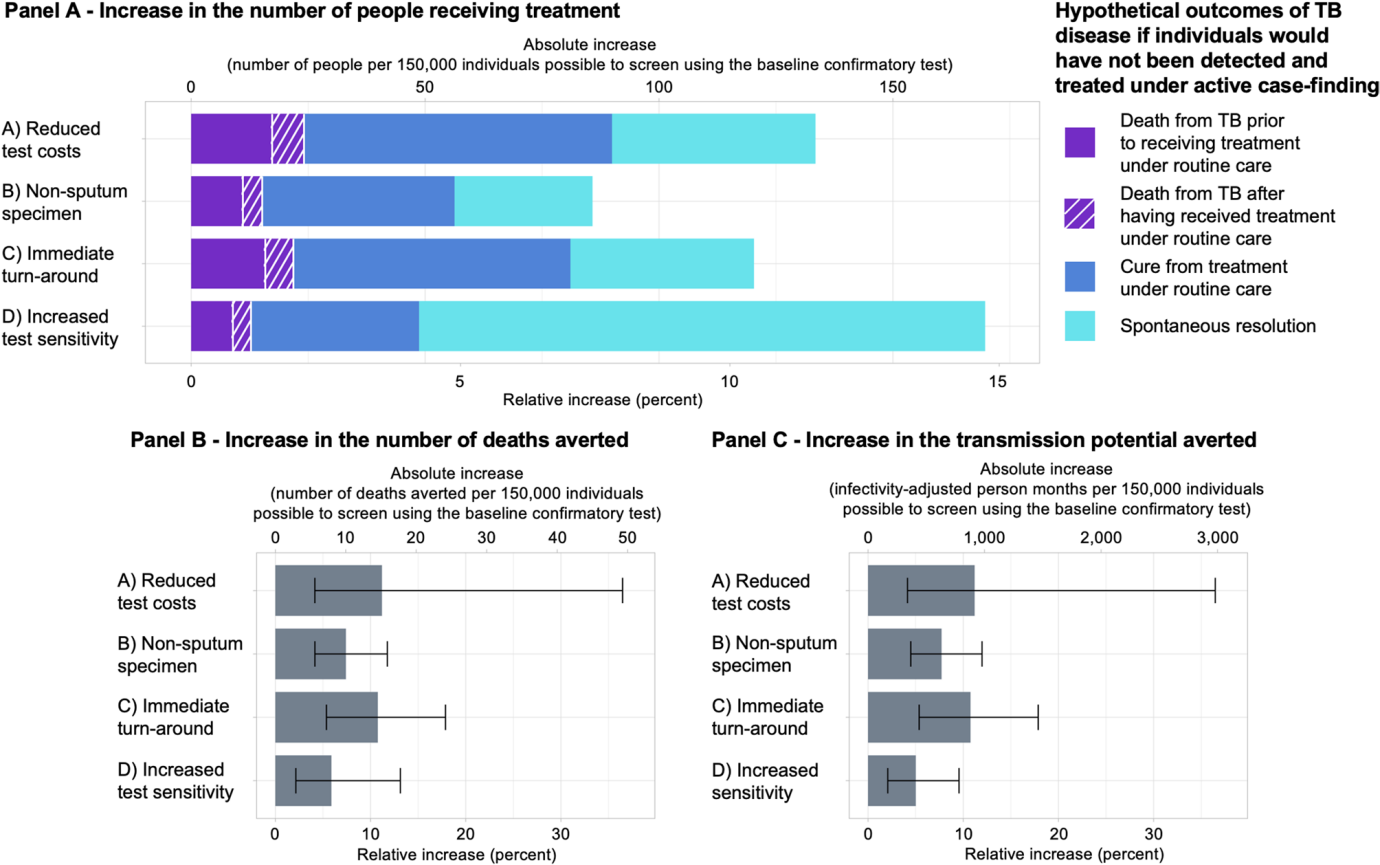
Epidemiological effect of an improved test to confirm tuberculosis, when used in an active case-finding campaign with a budget of 1 million US dollars. Bars show the potential epidemiological benefit of ACF using hypothetical improved confirmatory tests when screening a target population of 500,000 people for tuberculosis (TB) compared to conducting the same ACF efforts using Xpert Ultra to confirm a positive screening test result. An estimated 150,000 individuals could be screened under the allocated budget in the baseline comparator scenario. Test improvements considered are: (1) Increase in test sensitivity (from 69.0% to 80.0%; purple bar), using a non-sputum respiratory specimen type (increasing confirmatory specimen production from 93% to 100% for those eligible), (3) point-of-care testing (increasing the proportion of receiving the confirmatory test result from 91% to 100%), and (4) reduced costs (from $20 to $10 per confirmatory test). Panel **A** depicts the increase in the number of people with TB diagnosed and treated under ACF efforts when each of the named confirmatory test improvements is used compared to ACF utilizing the baseline confirmatory test. Herein, the purple area of the bars refers to people with TB who would have died in the absence of community-based screening, the dark blue area to people with TB who would have received treatment and been eventually cured even in the absence of ACF, and the light blue area to people in whom TB would have spontaneously resolved prior to receiving any treatment. Panel **B** shows the estimated reduction in TB mortality (number of TB-related deaths) and Panel **C** the projected reduction in TB transmission (infectivity-adjusted person-months) resulting from each of the test improvements

When considering mortality outcomes, the largest impact was found with reduced test costs (11% [4–36%] more deaths averted than through ACF with baseline confirmatory testing; 16 [5–44] incremental deaths averted), followed by immediate turn-around (11% [5–18%]; 14 [6–32]), using a non-sputum specimen (7% [4–12%]; 10 [4–21]), and increased test sensitivity (6% [2–13%]; 8 [3–19]). Relative results for averted transmission potential were similar to those for deaths averted. The greatest benefits resulted from reducing confirmatory test costs (11% [4–36%] more infectivity-adjusted person months averted than through ACF with baseline confirmatory testing; 949 [290-2,589] incremental infectivity-adjusted person months averted) or from immediate turn-around of test results (11% [5–18%]; 860 [350-1,898]). These reductions were similar when only transmission from individuals with high bacillary load was considered: 11% [4–36%] from reduced confirmatory test costs and 11% [5–18%] from immediate turn-around of test results (Fig. 3; Table 3).

**Table 3:** Epidemiological outcomes under active case finding for tuberculosis, using alternative tests to confirm positive screening results

Strategy	Routine care alone	Baseline active case-finding efforts	Active case-finding using an improved confirmatory test			
			Increased test sensitivity^a^	Non-sputum specimen^a^	Immediate turn-around of results^a^	Reduced test costs^a^
Total population						
Total number of people modeled	500,000 (500,000; 500,000)	500,000 (500,000; 500,000)	500,000 (500,000; 500,000)	500,000 (500,000; 500,000)	500,000 (500,000; 500,000)	500,000 (500,000; 500,000)
People with TB	8,029 (6,634; 9,380)	8,029 (6,634; 9,380)	8,029 (6,634; 9,380)	8,029 (6,634; 9,380)	8,029 (6,634; 9,380)	8,029 (6,634; 9,380)
People with TB receiving treatment	3,391 (2,588; 4,330)	3,927 (3,068; 4,918)	4,064 (3,178; 5,079)	3,968 (3,104; 4,968)	3,985 (3,119; 4,998)	3,998 (3,138; 4,988)
People with TB cured following treatment	3,065 (2,339; 3,914)	3,587 (2,805; 4,489)	3,722 (2,908; 4,659)	3,628 (2,836; 4,539)	3,644 (2,852; 4,555)	3,658 (2,866; 4,559)
People who die due to TB	1,136 (789; 1,586)	996 (701; 1,389)	987 (694; 1,377)	985 (694; 1,373)	980 (690; 1,368)	978 (691; 1,360)
People who die due to TB without receiving treatment	808 (484; 1,241)	651 (390; 1,018)	641 (383; 1,006)	639 (382; 1,004)	635 (378; 995)	632 (379; 991)
People in whom TB spontaneously resolves	3,777 (2,941; 4,728)	3,394 (2,618; 4,296)	3,261 (2,507; 4,160)	3,359 (2,592; 4,261)	3,349 (2,584; 4,253)	3,344 (2,585; 4,226)
People included in active-case finding (ACF) efforts						
People screened as part of ACF	n/a	149,811 (90,834; 217,928)	149,811 (90,834; 217,928)	149,811 (90,834; 217,928)	149,811 (90,834; 217,928)	167,583 (116,947; 235,578)
People with TB screened as part of ACF	n/a	2,382 (1,411; 3,642)	2,382 (1,411; 3,642)	2,382 (1,411; 3,642)	2,382 (1,411; 3,642)	2,676 (1,794; 3,932)
People with TB receiving treatment following ACF	n/a	1,151 (676; 1,813)	1,328 (784; 2,080)	1,242 (725; 1,951)	1,278 (754; 2,003)	1,297 (849; 1,969)
People with high bacillary load TB receiving treatment following ACF	n/a	735 (430; 1,154)	743 (435; 1,166)	793 (462; 1,239)	817 (479; 1,277)	829 (544; 1,250)
People with TB cured following treatment under ACF	n/a	1,077 (633; 1,702)	1,249 (735; 1,955)	1,162 (678; 1,830)	1,196 (704; 1,878)	1,213 (794; 1,848)
People with high bacillary load TB cured following treatment under ACF	n/a	673 (393; 1,056)	680 (398; 1,069)	725 (423; 1,135)	747 (438; 1,168)	759 (498; 1,143)
People cured under ACF who would have died under routine care	n/a	135 (64; 249)	143 (68; 263)	145 (68; 269)	150 (71; 278)	154 (78; 272)
Total transmission potential						
Cumulative infectivity-weighted person-months	44,978 (29,013; 66,784)	36,496 (23,456; 55,063)	36,051 (23,045; 54,577)	35,817 (23,027; 54,100)	35,562 (22,796; 53,780)	35,396 (22,846; 53,276)

^a^ See Table 1 for details on each confirmatory test improvement’s mechanism of benefit

### Oral-swab based testing

To match the mortality impact of baseline sputum confirmatory testing (assuming 93% of the study population to be able to produce sputum), for an oral-swab based test with reduced sensitivity, reduced test costs, and increased receipt of test results, our model estimated that oral-swab based testing would need to be between 61% and 100% as sensitive as the sputum-based confirmatory test (median estimate 80%). This sensitivity requirement fell to a median 65% [51–85%] and 54% [43–68%] when assuming lower (83% and 73%, respectively) sputum production. Results were similar when considering transmission potential averted as a metric (Figure S1).

### Sensitivity and scenario analysis

Neither variation of TB or HIV burden or tests costs in one-way sensitivity analyses (Text S6 and Figure S2) nor consideration of treatment costs as part of the ACF budget (Text S6 and Figure S4) materially affected the relative importance of different confirmatory test improvements. When including treatment costs, specificity also remained less influential than other confirmatory test characteristics (Figure S4). However, when spontaneous TB resolution was eliminated from the model, increased sensitivity became the confirmatory test characteristic leading to the greatest reductions in TB mortality and transmission potential (Text S6 and Figure S3). Assuming chronic-cough-based rather than radiographic screening (i.e., screening at lower cost, sensitivity and specificity), the reach of screening increased to 270,617 individuals (126,013–399,247; baseline scenario: 149,811 [90,834 − 217,928]), almost doubling the number of deaths averted to 234 (93–470; baseline scenario: 135 [64–249]) and infectivity-adjusted person-months averted to 14,316 (6,017–27,471; baseline scenario: 8,169 [4,182 − 15,020]). Under this scenario, the benefit of reducing test costs grew further, leading to a 21% (9–55%) increase in incremental TB deaths and incremental TB transmission potential averted (Text S6 and Figure S5). Assuming reduced sputum expectoration amongst individuals referred for confirmatory testing, using a non-sputum confirmatory test became the most relevant test improvement both in regards to increases in treatment referral as well as reductions in TB mortality and transmission (Text S6 and Figure S6). Assuming reduced linkage to treatment did not materially affect our model’s results (Text S6 and Figure S7). Keeping all other model parameters the same as in the baseline analysis, but improving the sensitivity, cost, specimen-type, and turn-around time of the confirmatory test simultaneously, the reach of ACF increased to 167,583 (116,947 − 235,578) individuals. This allowed ACF to avert 58 (30–103; 42% [26–76%]) more deaths and 3,413 (1,830-6,024; 41% [25–75%]) more infectivity-weighted person months averted than through ACF with the baseline confirmatory test (Text S6).

## Discussion

This modeling analysis evaluated the potential epidemiological impact of improvements to current tools for confirmatory testing in community-based screening (i.e., ACF) for TB. Although accuracy is often prioritized in development of TB diagnostic tests, we predict reduced test costs, immediacy of turn-around, and the use of a highly-available specimen type to have greater impact on mortality and transmission potential, when considering a confirmatory testing use case during TB screening of high-TB-prevalence community. However, none of these improvements in isolation increased the mortality or transmission impact of ACF by more than 11%; thus, a greater variety of enhancements to feasibility and effectiveness are needed if ACF is to play a major role in meeting WHO End TB targets [56].

Our results indicate that an increase in confirmatory test sensitivity (from 69% to 80%) would result in the largest increase in the number of people diagnosed and treated through ACF, but would yield only small benefit in terms of reducing TB mortality and transmission. This discrepancy occurs because the people incrementally diagnosed by a more sensitive test would have lower bacillary loads; besides having lower current infectivity [11, 57], survival data from the pre-antibiotic data suggest such individuals have lower TB mortality risk [58], and models also translate this into less cumulative future transmission [12]. By contrast, reducing test costs or operational barriers to confirmatory testing, e.g., with a point-of-care test, would enable detection of additional people with different kinds of TB, including both low and high bacillary load. By also increasing detection of high bacillary-load TB, i.e., more fatal and more transmissible TB, lowering test costs or operational barriers is estimated to have a larger impact on mortality and transmission outcomes than further increasing test sensitivity. Hence, focusing efforts on developing less expensive and easier to use confirmatory tests might achieve greater population health benefits than further increasing test sensitivity. These results are consistent with recent modeling showing that greater accessibility may be more impactful than high sensitivity in clinical diagnostic settings [59]. Also, they are aligned with current WHO target product profiles for TB diagnostics, requiring a lower minimal sensitivity for non-sputum point-of-care tests compared to high-complexity sputum tests [10] – strengthening the case for focusing diagnostics development efforts on improving cost and ease of use.

The relative importance of sensitivity or cost characteristics could change under certain circumstances. Our primary analyses assume that community-based active case-finding would use some of the most accurate screening tools currently available, i.e., chest X-ray. However, if a less specific screening tool, such as symptom screening [15], is used, more people will require confirmatory testing, and confirmatory testing will comprise a greater proportion of the overall ACF budget. As seen in our scenario analysis, this increases the importance of confirmatory test costs relative to operational improvements in determining intervention reach and impact. In addition, as seen in our sensitivity analysis, including a population with different distributions of symptom status, HIV status, and bacillary load – as might be found in countries other than Uganda, or with different approaches to selecting individuals for ACF – can affect the absolute impact of the confirmatory test improvements. However, we did not find it to largely change their relative importance. Furthermore, certain test improvements might require counterbalancing reductions in sensitivity. For example, we estimate that oral-swab-based testing – the currently most promising technique for complementing sputum testing – would require at least 65–80% the sensitivity of sputum-based testing to achieve a similar population health benefit in a setting where 83–93% of individual are able to produce sputum. Most recent studies of tongue swab testing among symptomatic individuals presenting for care suggest sensitivities at least this high [37, 38]. If sensitivity at this level can be achieved in the context of community-based screening, oral-swab samples might provide a valuable addition to sputum-based testing for confirmatory testing in ACF campaign. In contrast, other methods for complementing sputum Xpert-Ultra – like sputum smear-microscopy – can offer similar reductions in costs and turnaround time as oral-swab based testing. However, with sputum smear-microscopy’s sensitivity likely falling below the estimated 65–80% threshold [60], ACF using smear-microscopy as the confirmatory test might not achieve a similar impact as ACF using Xpert Ultra.

We estimated that isolated improvements to confirmatory tests were likely to increase the overall mortality and transmission impact of ACF by no more than 11%. In the context of population-wide systematic screening, this incremental benefit could be comparable to, for example, the estimated benefit of hypothetically improved TB treatment regimens (assuming 99% efficacy and 2 months treatment duration) [24]. Given expected synergies between different test improvements (e.g., if a non-sputum specimen were available, more individuals could complete confirmatory testing, further increasing the benefit of immediate results turn-around), improving all of the modeled test characteristics was estimated to increase the effect of ACF on TB mortality and transmission potential by 42%. However, such an improved confirmatory test is unlikely to be available in foreseeable future. Therefore, to optimally enhance the impact and cost-effectiveness of community-based active case-finding in shorter time-frame, other improvements than confirmatory testing should be pursued as well; possibilities include better tools for identifying high-risk populations or simpler and more affordable screening tests for specific use-cases, such as C-reactive protein testing, which has shown reasonable sensitivity in individuals with high bacillary load [61]. Thus, while changes through improved confirmatory testing could meaningfully increase the effectiveness and cost-effectiveness of ACF, especially if multiple improvements could be combined, other improvements than confirmatory testing should also be considered to improve ACF within due time.

Our results are limited by simplifying assumptions in our model’s representation of TB disease states, case-finding campaigns, and treatment outcomes. Because it is not ethical to perform observational studies of the untreated TB disease course, disease durations and outcomes must be inferred from cross-sectional and historical data. Thus, our estimates of the outcomes of prevalent TB under routine care, which we based on prior modeling analyses, are subject to those models’ uncertainties, including data limitations (e.g., on HIV-associated TB), reliance on historical microbiological classifications, and uncertain accuracy of country-level TB notification and mortality tabulations [12, 39]. Our estimates of Xpert Ultra accuracy among modeled subpopulations of prevalent TB are likewise limited by scarcity of data on the performance of molecular testing relative to culture in a screening context and on its correlation with screening chest X-ray. In modeling the case-finding intervention, we assumed improved test characteristics would strengthen the care cascade, but ability to realize these gains also depends on test implementation. In addition, because we represented ACF cross-sectionally, we do not capture second order effects that transmission-relevant confirmatory test improvements might have on disease burden and available resources over sufficiently long budgetary timelines. Moreover, we have focused on how improvements to diagnostic tests would affect the outcomes of community-wide screening, but it is likely that those improvements would also enhance other TB interventions (e.g., contact screening and prevention) in ways we have not modelled. Furthermore, we dichotomize more nuanced population characteristics such as HIV severity, presence of chronic cough, and bacillary load. Lastly, our modeling of treatment outcomes is simplified by not explicitly modeling re-treatments, drug resistance, or relationships between the timing of diagnosis and treatment outcomes.

In conclusion, we found that reducing operational barriers for confirmatory TB testing – such as changing to a non-sputum specimen or facilitating immediate turn-around of test results – is likely to lead to greater impact on TB transmission and mortality in the context of community-based ACF than increasing sensitivity. Since improvements in confirmatory tests are likely to have modest epidemiological impact in isolation, other measures to improve ACF should be explored as well.

## Supplementary Information

Supplementary Material 1

Supplementary Material 2

Supplementary Material 3

## Data Availability

All data generated or analyzed during this study are included in this published article (and its supplementary information files).

## Abbreviations

ACF: Active case–finding
HIV: Human immunodeficiency virus
TB: Tuberculosis
UR: Uncertainty range
USD: United states dollars
WHO: World health organization
